# *Clock *mutation affects circadian regulation of circulating blood cells

**DOI:** 10.1186/1740-3391-4-13

**Published:** 2006-10-02

**Authors:** Katsutaka Oishi, Naoki Ohkura, Koji Kadota, Manami Kasamatsu, Kentaro Shibusawa, Juzo Matsuda, Kazuhiko Machida, Shuichi Horie, Norio Ishida

**Affiliations:** 1 Clock Cell Biology Research Group, Institute for Biological Resources and Functions, National Institute of Advanced Industrial Science and Technology (AIST), Central 6, 1-1-1 Higashi, Tsukuba, Ibaraki 305–8566, Japan; 2 Clinical Molecular Biology, Faculty of Pharmaceutical Sciences, Teikyo University, 1091-1 Suarashi, Sagamiko, Tsukui, Kanagawa 199–0195, Japan; 3 Agricultural Bioinformatics Research Unit, Graduate School of Agricultural and Life Sciences, The University of Tokyo, 1-1-1 Yayoi, Bunkyo-ku, Tokyo 113–8657, Japan; 4 Department of Hygiene and Public Health, School of Human Sciences, Waseda University, 2-579-15 Mikajima, Tokorozawa, Saitama 359–1192, Japan; 5 Graduate School of Life and Environmental Sciences, University of Tsukuba, Tsukuba, Ibaraki 305–8502, Japan

## Abstract

**Background:**

Although the number of circulating immune cells is subject to high-amplitude circadian rhythms, the underlying mechanisms are not fully understood.

**Methods:**

To determine whether intact CLOCK protein is required for the circadian changes in peripheral blood cells, we examined circulating white (WBC) and red (RBC) blood cells in homozygous *Clock *mutant mice.

**Results:**

Daytime increases in total WBC and lymphocytes were suppressed and slightly phase-delayed along with plasma corticosterone levels in *Clock *mutant mice. The peak RBC rhythm was significantly reduced and phase-advanced in the *Clock *mutants. Anatomical examination revealed hemoglobin-rich, swollen red spleens in *Clock *mutant mice, suggesting RBC accumulation.

**Conclusion:**

Our results suggest that endogenous clock-regulated circadian corticosterone secretion from the adrenal gland is involved in the effect of a *Clock *mutation on daily profiles of circulating WBC. However, intact CLOCK seems unnecessary for generating the rhythm of corticosterone secretion in mice. Our results also suggest that CLOCK is involved in discharge of RBC from the spleen.

## Background

The number of circulating white blood cells (WBC) involved in immune defense is subject to high-amplitude circadian rhythms [1,2]. Periodic changes in the number of leukocytes circulating in the peripheral blood might result from several factors. These include the distribution of circulating and marginal cell components of tissues and organs, influx from storage sites, cell proliferation, release of *de novo *cells into the circulation, and cell destruction and removal [2]. The underlying mechanisms of circadian changes in circulating blood cells have not been fully elucidated, although the numbers of monocytes, natural killer (NK) cells, T and B cells vary in a circadian manner [3-5].

*Clock *was the first clock gene identified in vertebrates using *N*-ethyl-*N*-nitrosourea mutagenesis screening [6]. *Clock *mutants exhibit abnormally long periodicity of behavior for the initial 5 to 15 cycles and subsequently lose circadian rhythmicity under constant darkness, although behavioral rhythms are completely entrained to environmental light under a light-dark cycle [6]. However, we reported that the long periodicity of *Clock *mutant mice on a Jcl:ICR background lasts for over one month under constant darkness [7,8]. The *Clock *gene encodes a basic helix-loop-helix (bHLH)-PAS transcription factor that plays an important role in the negative feedback loop of the circadian clock [9,10]. Like other bHLH transcription factors, CLOCK binds DNA and modulates transcription following dimerization. As the *Clock *allele is truncated and causes a deletion of 51 amino acids, the mutation presumably would not significantly affect the N-terminal bHLH and PAS domains, leaving CLOCK dimerization and DNA binding intact [9,10]. In fact, mutant CLOCK protein can still form heterodimers with BMAL1 (a bHLH-PAS transcription factor) that binds to DNA, but these are deficient in transcriptional activity [9,10]. CLOCK protein is involved in the transcriptional regulation of several circadian output genes as well as in the core circadian clock [11,12].

To determine whether intact CLOCK protein is required for the circadian changes in peripheral blood cells, we examined circulating WBC and red blood cells (RBC) and evaluated plasma levels of corticosterone (CS) in homozygous *Clock *mutant mice.

## Methods

### Animals

*Clock *mutants were derived from mice supplied by J.S. Takahashi (Northwestern University, Evanston, IL.). The mice originally had the *Clock *allele on a BALB/c and C57BL/6J background. A breeding colony was established by further backcrossing with Jcl:ICR mice and the new strain was subsequently maintained by interbreeding for at least 10 generations [13]. Genotypes were determined using PCR as described [14]. The mice were studied at 8–10 weeks of age. Mice were housed under a 12 h light-12 h dark cycle [LD 12:12; lights on at 0:00 h]. A white fluorescent lamp was used as a source of light during the day (150–200 lux at the level of the cages).

### Analysis of peripheral circulating blood cells

To determine peripheral circulating blood cells at each time point and genotype, mice were killed by decapitation, trunk blood was collected into heparinized capillary tubes, blood cells were automatically counted (Sysmex F-820 Blood Counter, Toa Medical Electron Inc., Japan), and the differential was determined by Wright-Giemsa staining followed by light microscopy.

### Plasma corticosterone (CS) and erythropoietin (EPO)

To determine CS and EPO at each time point and genotype, mice were killed by decapitation and trunk blood was collected into heparinized capillary tubes. Blood was collected in the dark under a dim red lamp to avoid any possible influence of light on the corticosterone profile [15]. Plasma was immediately separated from blood samples by centrifugation at 3,000 rpm for 10 min at 4°C and stored at -80°C. Mouse plasma CS and EPO levels were determined using an EIA kit for rat CS (Diagnostic Systems Laboratories, Inc.) and an ELISA kit for mouse EPO (R&D Systems, Minneapolis, MN), respectively.

### Splenic hemoglobin (HGB) content

The spleen was homogenized in distilled water. After centrifugation at 3,000 rpm for 15 min, HGB concentrations of the supernatants were determined using the Hemoglobin B-test Wako (Wako Pure Chemical Industries Ltd., Osaka, Japan).

### Statistics

We used a nonlinear least-squares (NLLS) Marquardt-Levenberg algorithm to fit a curve to the observations, and determined the acrophase (the timing of the cosine maximum) of numbers of total white blood cells, lymphocytes, and red blood cells, hemoglobin and corticosterone levels. We defined the function as f(x) = M + A cos(2p/P*(x – T)) and set the variables M (MESOR; mean statistics of rhythm), A (Amplitude) and T (Acrophase) as the fit parameters. P was set at 24, because the present study proceeded under LD 12:12. Acrophases were compared using Welch's t-test, and *p *< 0.05 was considered statistically significant. All values are expressed as means ± SEM. Scheffé's multiple comparison tests assessed specific differences between genotypes.

## Results

**Figure 1:**
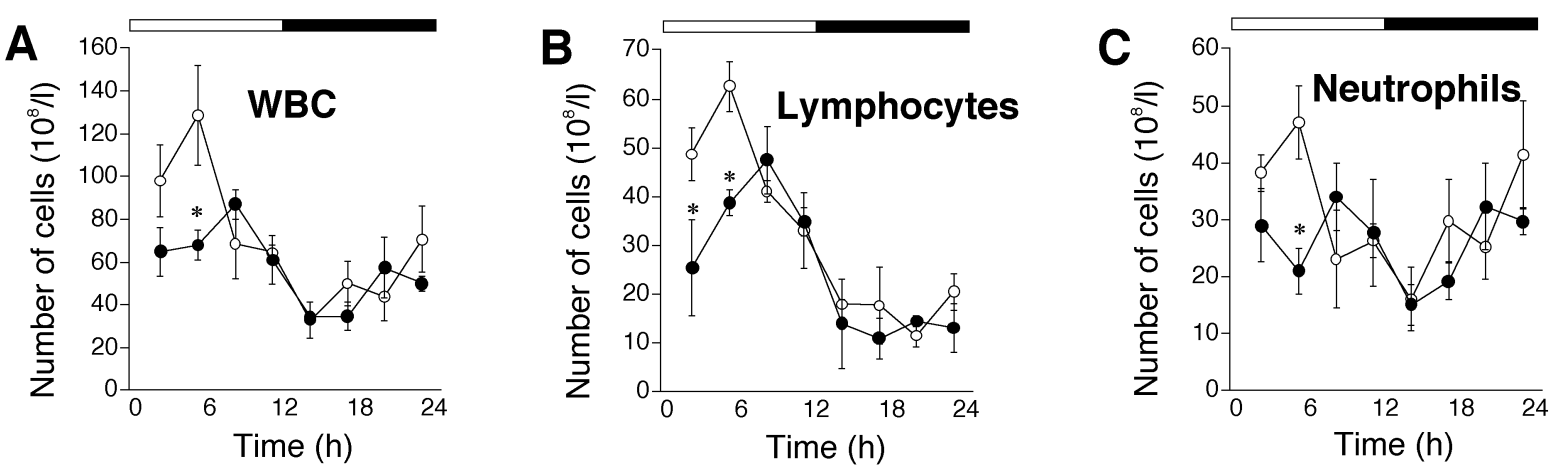
**Circadian variations in peripheral circulatingleukocytes in *Clock *mutant mice**. (A) Total number of white blood cells (WBC), (B) number of lymphocytes, (C) number of neutrophils. Open and filled circles, values from wild-type and *Clock *mutant mice, respectively. Open and solid bars, lights on and off, respectively. Values represent means ± SEM (n = 3). Significant differences between genotypes are shown as *P < 0.05.

Figure 1A shows that the number of total WBC fluctuated in a circadian pattern that peaked during the early morning both in wild-type and in *Clock *mutant mice, although the acrophase of the rhythm was delayed from 3.7 ± 1.3 h in wild-type mice to 5.5 ± 0.7 h in the *Clock *mutants. The acrophase of the lymphocyte rhythm was also significantly delayed from 4.9 ± 1.1 h in wild-type mice to 7.3 ± 0.5 h in the *Clock *mutants (Fig. 1B). The temporal pattern of number of neutrophils in *Clock *mutant mice was bimodal, whereas the pattern of lymphocytes was similar to that in wild-type mice (Fig. 1C). The *Clock *mutation reduced the numbers of total WBC, lymphocytes and neutrophils during the light period (Fig. 1A – C).

**Figure 2:**
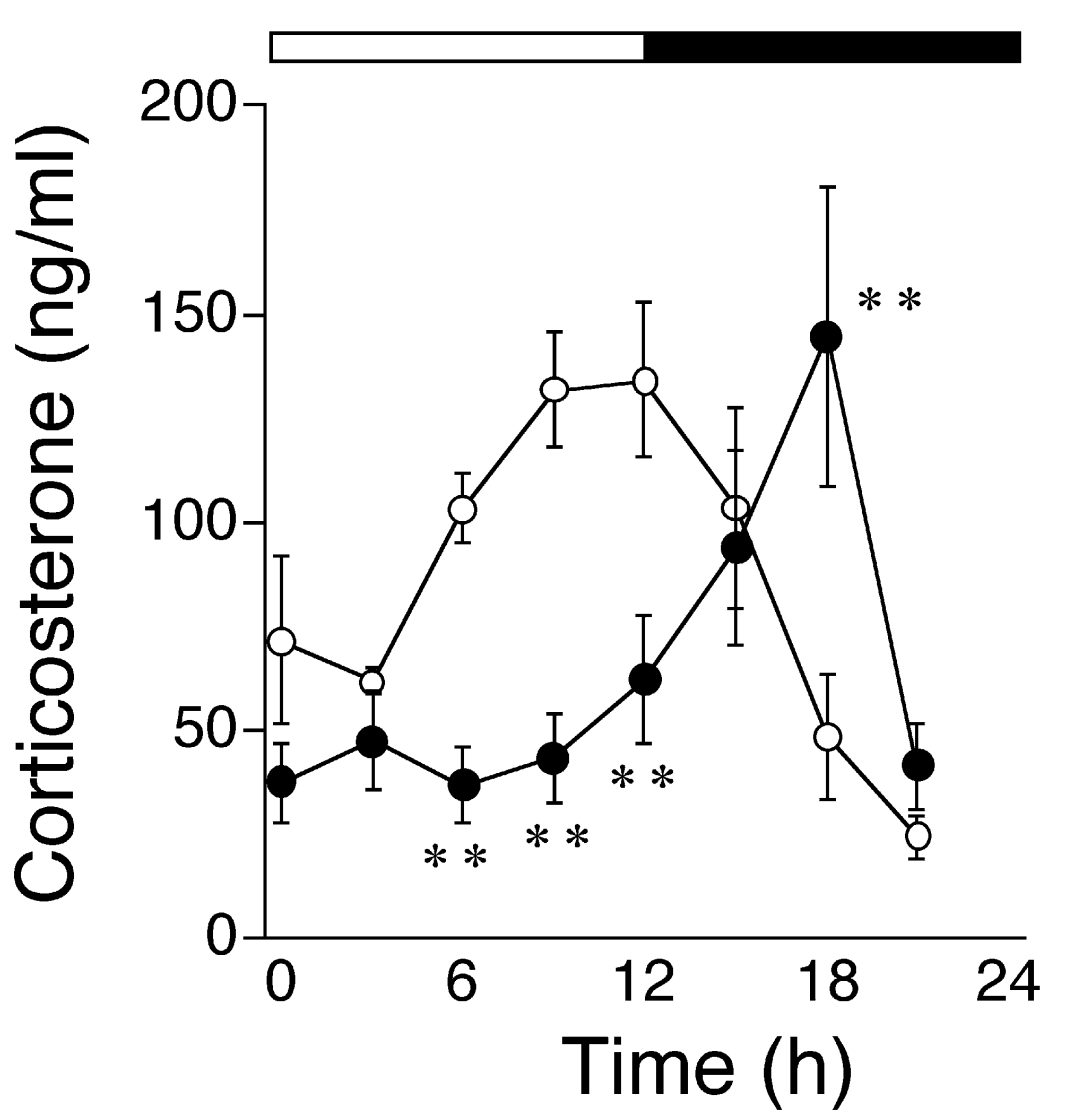
**Circadian variations in plasma corticosterone (CS) levels in *Clock *mutant mice**. Open and filled circles indicate values from wild-type and *Clock *mutant mice, respectively. Open and solid bars represent lights on and off, respectively. Values represent means ± SEM (n = 4). Significant differences between genotypes are shown as **P < 0.01.

Figure 2 shows that the plasma levels of CS fluctuated in a circadian manner in both wild-type and *Clock *mutant mice, but the acrophase significantly differed between the genotypes (*p *< 0.01). In wild-type mice, plasma CS peaked at 9.8 ± 0.4 h, just before the light-dark transition [4]. In contrast, plasma CS levels in *Clock *mutant mice peaked at 16.4 ± 0.3 h, the middle of the dark period. The peak CS levels were similar in the two genotypes.

**Figure 3:**
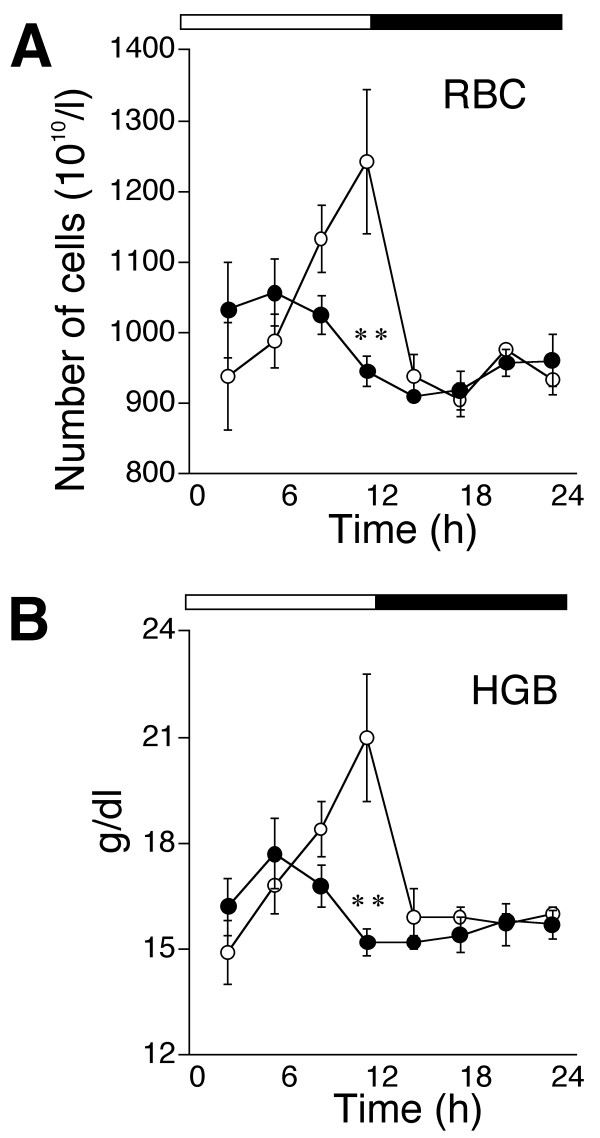
**Circadian variations in peripheral circulating redblood cells (RBC) in *Clock *mutant mice**. (A) Number of RBC and (B) blood levels of hemoglobin (HGB). Open and filled circles, values from wild-type and *Clock *mutant mice, respectively. Open and solid bars represent lights on and off, respectively. Values represent means ± SEM (n = 3). **P < 0.01, significant difference between genotypes.

Figure 3A shows that the number of RBC peaked at 9.5 ± 0.3 h. just before the light-dark transition in wild-type mice. However, the amplitude was reduced and the acrophase was significantly advanced to 4.5 ± 1.0 h (*p *< 0.05) in *Clock *mutant mice (Fig. 3A). Blood levels of HGB closely correlated with the number of RBC (Fig. 3). The acrophase of HGB was 10.0 ± 0.4 h in wild-type mice and 4.5 ± 1.0 h in the *Clock *mutants (*p *< 0.05).

Plasma EPO levels were slightly but significantly higher in *Clock *mutant (77.2 ± 0.09 pg/l) than in wild-type (68.8 ± 0.07 pg/l) mice (*p *< 0.01). Plasma EPO levels were not rhythmic in either genotype (data not shown).

**Figure 4:**
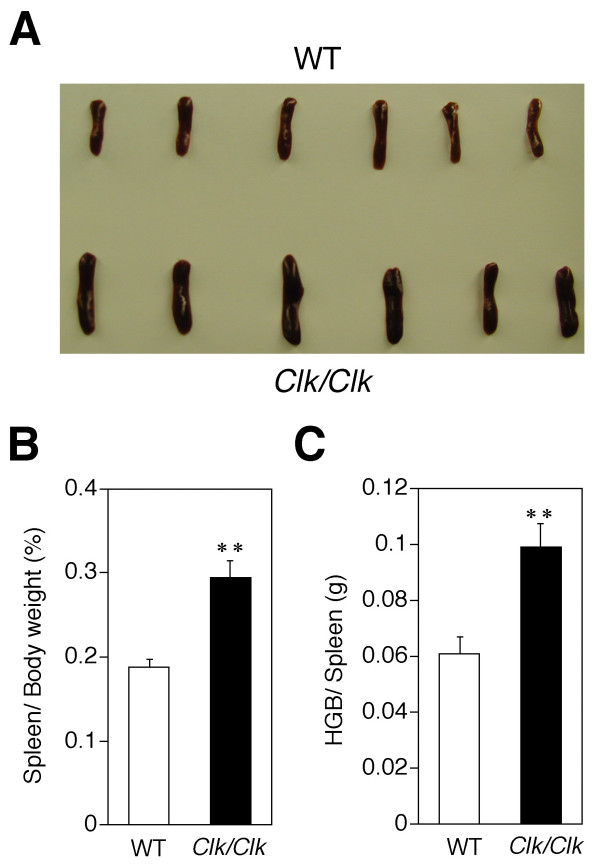
**Swollen red spleens from *Clock *mutant mice**. (A) Spleens from wild-type and *Clock *mutant mice. (B) Graphs show ratio (%) of wet weight of spleen per body weight in wild-type and *Clock *mutant mice. (C) Graphs show hemoglobin (HGB) content in wild-type and *Clock *mutant mice. Values represent means ± SEM (n = 6). **P < 0.01, significant difference between genotypes.

Anatomical examination of *Clock *mutant mice revealed a swollen red spleen (Fig. 4A). The wet weight of the spleen per body weight of *Clock *mutants was about 1.5-fold greater than that of wild-type mice (*p *< 0.01) (Fig. 4B). The splenic HGB content was also significantly increased in *Clock *mutant mice (*p *< 0.01) (Fig. 4C).

## Discussion

The circadian rhythm in the number of circulating blood cells in mammals is highly regular and reproducible [16]. However, the complex nature of this regulation has prevented elucidation of the underlying mechanisms of circadian changes in circulating blood cells [2]. The present study found that a homozygous mutation of the circadian clock gene, *Clock*, affects the circadian fluctuation of circulating WBC and RBC in mice. This finding suggests that the circadian rhythm in the number of blood cells is dependent on core components of the circadian clock.

Steroid hormones play important roles in generating the circadian rhythm of circulating blood cells [17]. Patients with an adrenal insufficiency or with adrenal hyperplasia have low and high endogenous cortisol levels together with high and low lymphocyte counts, respectively [18,19]. Corticosteroids cause the efflux of some lymphocytes from the vasculature and their retention in the lymphatic circulation [20]. The mechanism through which corticosteroids cause such movements may involve the expression of cell adhesion molecules (CAMs) [21,22]. The expression levels of leukocytes (including lymphocytes, neutrophils, and monocytes) and of CAMs are highest when plasma cortisol concentrations are maximal [23]. Interestingly, we previously demonstrated that mRNA expression levels of *ICAM1 *are reduced in *Clock *mutant mice [11]. The present study found that the acrophase of total WBC and lymphocytes numbers was delayed for few hours in accordance with that of plasma CS levels in *Clock *mutant mice. We previously reported that the acrophase of other physiological parameters such as body temperature (17.2 ± 0.2 h and 19.9 ± 0.3 h in wild-type and *Clock *mutants, respectively) and spontaneous activity (17.5 ± 0.4 h and 19.9 ± 0.3 h in wild-type and *Clock *mutants, respectively) is also delayed in *Clock *mutant mice [13]. Our present results suggested that circadian clock-regulated diurnal CS secretion from the adrenal gland is involved in the effect of the *Clock *gene mutation on circadian changes in the numbers of peripheral circulating WBC.

The circadian fluctuation of plasma CS concentration is blunted at low levels in *Clock *mutant mice with a CBA/6CaH background under LD conditions [24]. However, the present study identified a circadian CS rhythm in *Clock *mutant mice with a Jcl:ICR background under LD conditions, although the acrophase was delayed in mutant mice. These remarkable strain differences of the effect of a *Clock *gene mutation on circadian CS secretion are very similar to those on behavior, as the circadian behavior rhythm of homozygous *Clock *mutant mice is completely abolished under constant darkness in both C57BL/6J [6] and CBA/6CaH [25] backgrounds but maintained for at least 2 months in the Jcl:ICR background [7]. A recent study using the *Cre-LoxP *system found that CLOCK is not essential for generating the circadian locomotor rhythm in mice [26]. Mutant CLOCK protein might provoke gain-of-function effects in the mutant mice, because the *Clock *allele is truncated and causes a deletion of the transactivation domain, which leaves DNA binding intact. The phenotypic differences (such as CS rhythm, locomotor activity [7], and metabolism [27]) between the *Clock *mutant mice with different backgrounds might reflect expression levels of the truncated CLOCK protein, which in the Jcl:ICR background would interfere less with circadian and other physiological processes. The present study demonstrated that intact CLOCK is not essential for either circadian CS secretion or the circadian fluctuation of circulating WBC in mice, at least under an LD condition.

On the other hand, a study of blood samples withdrawn over a period of 24 h has established a circadian rhythm in E-rosette-forming (T) cells *in vitro *that persists in 4-day-old cell cultures [28]. This finding suggests that fluctuations in some lymphocyte subpopulations depend on a cellular circadian oscillator [2]. We revealed that many circadian output genes, as well as those in the core circadian clock, are governed by CLOCK protein at the level of transcription [11]. In the present study, the circadian rhythm of circulating neutrophils was a damped bimodal process in *Clock *mutant mice, although that of lymphocytes was only phase-delayed. Granulocytes emigrate from the bloodstream to tissues and cannot recirculate, whereas lymphocytes continuously recirculate from tissues through the lymph back to the blood. Therefore, the *Clock *mutation might affect the proliferation of neutrophils in the bone marrow and/or neutrophil apoptosis in peripheral tissues.

We showed here that the circadian fluctuation of circulating RBC is regulated not only by the consequence of physiological rhythms such as feeding and sleeping but also by the endogenous circadian clock. The acrophase of the diurnal RBC rhythm is near the light-dark transition in nocturnal mice. The acrophase of circulating RBC in diurnally active humans is also at the time of the light-dark transition [16]. Thus, the relationship between circulating WBC of about 180° between circadian rhythms in diurnally active humans and nocturnally active rodents does not apply to the RBC rhythm. This resembles the expression of circadian clock genes in the suprachiasmatic nucleus (SCN), which is the master circadian pacemaker that controls most of the physiological circadian rhythms of mammals. The expression phase of clock genes (*Per1 *and *Per2*) in the SCN is almost identical between rodents that are active during the night [9,10] and during the day [29-31]. Thus, the SCN might tightly regulate the circadian phase of circulating RBC in mammals, whereas the phase of WBC seems to be affected by physiological rhythms such as those of feeding, locomotor activity and blood CS levels.

The amplitudes of RBC rhythms are very small and are of interest from a physiological viewpoint [32]. Circulating RBC numbers are determined not only by their clearance from the peripheral circulation in the spleen and liver but also by their production in bone marrow. Actually, circulating reticulocytes have a circadian rhythm, suggesting their circadian periodic release from the bone marrow [16]. The primary erythropoietic regulator EPO controls RBC production. We also found that plasma levels of EPO were slightly but significantly increased in *Clock *mutant mice throughout the day. Thus, the *Clock *mutation may affect the diurnal regulation of RBC production in the bone marrow.

The mean cell volume (MCV) of erythrocytes in the present study did not appear to exhibit circadian rhythmicity or to show genotypic differences. Figure 3 shows that HGB levels in the blood were closely correlated with the number of RBC. These results suggest that the availability of erythropoietic iron remained intact in the *Clock *mutant mice.

The lifespan of circulating RBC produced in bone marrow is determined by their elimination from the spleen. The RBC and HGB levels in the present study were still rhythmic and phase-advanced in *Clock *mutant mice, although the circadian rhythm of RBC and HGB in circulating blood was severely damped in the mutants. Anatomical examination revealed swollen red spleens and a significantly increased splenic HGB content in *Clock *mutant mice. These findings suggest that the effects of the *Clock *mutation on RBC rhythm results from the enhanced elimination of RBC from the bloodstream and/or a dysfunction in RBC discharge from the spleen.

Our results also indicated that the *Clock *mutation affects the circadian regulation of both blood CS levels and peripheral circulating blood cells, but that intact CLOCK is not essential to generate the rhythm. Further elucidation of the functions of circadian clock genes should reveal the underlying mechanisms of circadian changes in mammalian immune functions.

## Competing interests

The author(s) declare that they have no competing interests.

## Authors' contributions

KO and NO participated in data collection and drafted the manuscript. KK performed a circadian phase analysis. MK discovered the swollen red spleens in *Clock *mutant mice. KS and SY helped with data collection. JM, KM, SH, and NI supervised the study. All authors read and approved the final version of the article.
